# Association Between Leisure-Time Physical Activity and Self-Reported Hypertension Among Brazilian Adults, 2008

**DOI:** 10.5888/pcd10.130032

**Published:** 2013-10-24

**Authors:** Lilian G. Perez, Michael Pratt, Eduardo J. Simoes, Lenildo de Moura, Deborah C. Malta

**Affiliations:** Author Affiliations: Michael Pratt, Centers for Disease Control and Prevention, Atlanta, Georgia; Eduardo J. Simoes, University of Missouri School of Medicine, Columbia, Missouri; Lenildo de Moura, Deborah C. Malta, Ministry of Health of Brazil, National Coordination of Non-Communicable Diseases, Brasília, Federal District, Brazil.

## Abstract

**Introduction:**

Physical inactivity is a risk factor for hypertension. The objective of this study was to examine the association between self-reported leisure-time physical activity and hypertension among Brazilian adults categorized by sex and body weight.

**Methods:**

The study used data from adult respondents in 26 capital cities and the Federal District to VIGITEL (N = 54,353), Brazil’s 2008 national surveillance system for risk and protective factors for chronic diseases. We conducted a multivariate logistic regression analysis to investigate the association between self-reported leisure-time physical activity and hypertension and examined whether sex or body weight modified this relationship.

**Results:**

The prevalence of self-reported hypertension was high among women, older people, and people with fewer years of education. Overall, leisure-time physical activity decreased with increasing age, increased with increasing education level, and was higher among men than women. The association for leisure-time physical activity and hypertension was modified by sex but not body weight. Leisure-time physical activity reduced the odds of hypertension in men.

**Conclusion:**

On the basis of self-reporting, leisure-time physical activity may be protective against hypertension in Brazilian men. Inclusion of other physical activity domains in the analyses may be necessary to fully understand the complex relationship between physical activity and hypertension by sex. In addition, public health priorities in Brazil for improving physical activity can target the entire population and not just those who are overweight or obese.

## Introduction

Physical inactivity is a leading risk factor for disease and death and thus accounts for more than 5 million deaths worldwide each year (1). Regular participation in physical activity can reduce the risk of adverse health outcomes and improve chronic health conditions (2,3). Despite recognition of the health benefits of regular physical activity, surveillance data from various countries show that physical activity levels remain low (4). Such data pertain almost exclusively to high-income countries, where surveillance systems are well established. Only recently have a handful of low-and-middle income countries developed national surveillance systems for noncommunicable chronic diseases (NCDs) and their risk factors, including Brazil’s Sistema de Vigilância de Fatores de Risco e Proteção para Doenças Crônicas por Inquérito Telefônico (VIGITEL — Telephone-based Surveillance of Risk and Protective Factors for Chronic Diseases), a telephone-based system established in 2006 and based on the US Behavioral Risk Factor Surveillance System (BRFSS) (5).

The proportion of physically inactive people in Brazil has decreased slightly in recent years but remains consistently higher for women than men in the leisure-time, occupation, and transport physical activity domains, but not for household chores (6). The prevalence of self-reported hypertension in Brazil is higher in women than men (5) and increases with age and body mass index (BMI) (7).

Although VIGITEL has improved surveillance of NCDs and their risk factors in the urban population in Brazil, limited research has examined the association between leisure-time physical activity (LTPA) and hypertension and tried to determine whether this relationship is modified by sex or body weight. The objective of this study, therefore, was to examine the association between self-reported LTPA and hypertension among Brazilian adults according to sex and body weight by using multivariate logistic regression on VIGITEL 2008 data.

## Methods

### Study population

This study was cross-sectional and involved data collected in the 2008 VIGITEL system. The system collected self-reported data from a nationally representative probabilistic sample of adults (aged ≥18 years) living in households with telephones in 27 cities (each capital of the 26 Brazilian states and the Federal District) (N = 54,353) (5). Details on the sampling methodology and data collection are described elsewhere (5).

### Measures

Practice of LTPA was based on the World Health Organization (WHO) definition (8) used by the Brazilian Ministry of Health in 2008. For adults aged 18 to 64 years, being active during leisure time was defined as engaging in light or moderate physical activity (eg, walking, body building, gymnastics, swimming, martial arts, biking) for at least 30 minutes daily on 5 or more days a week or intense physical activity (eg, running, aerobics, soccer, basketball) for at least 20 minutes daily on 3 or more days a week, during leisure time in the past 3 months. On the basis of this definition, people were categorized as meeting the LTPA recommendations or not meeting the recommendations. We used participants’ self-reported weight and height to estimate BMI and to categorize them as normal weight (BMI <25 kg/m^2^) or overweight or obese (BMI ≥25 kg/m^2^) (9,10). People with self-reported hypertension were those who replied yes to the question, “Has a physician ever diagnosed you with high blood pressure?”

### Statistical analysis

Data management and analyses were carried out by using computer software program SAS version 9.2 (SAS Institute Inc, Cary, North Carolina). The final analyses used a weight variable to partially account for households not covered by the universal landline telephone network in Brazil (response rate, 74.6%). Calculation of this weight is discussed in detail in the 2008 VIGITEL report (5).

To examine the association between LTPA and self-reported hypertension, we developed a multivariate logistic regression model using hypertension as the outcome variable and based significance on a 5% significance level (*P* < .05) and 95% confidence intervals (CIs). We adjusted the model for the following predictor variables: continuous variables — age (in years), BMI (in kg/m^2^), and education (in years); dichotomous variables — sex (male or female) and self-report of a physician diagnosis of diabetes mellitus (yes or no), cardiac event (yes or no), and dyslipidemia (yes or no). To examine whether sex and body weight modified the association between LTPA and hypertension, we used the final model to obtain adjusted odds ratios (ORs) for the following groups: normal weight men, overweight men, normal weight women, and overweight women.

### Ethical considerations

Because of the VIGITEL system design (ie, telephone-based interviews), written free informed consent was not required, and oral consent was obtained at the time of the telephone interview with the respondents. The study was approved by the National Human Research Ethics Committee of the Brazilian Ministry of Health.

## Results

Slightly more than 50% of the overall sample were women, more than 65% were aged 18 to 44 years, and approximately 85% had completed 12 years or less of schooling (Table 1). Approximately 44% of respondents were categorized as overweight or obese. The prevalence of self-reported chronic conditions was 23.9% for hypertension, 5.5% for diabetes, 16.8% for dyslipidemia, and 2.8% for a cardiac event.

**Table 1:** Characteristics of All Adult (≥18 Years) Respondents (N = 54,353) and Those With Self-Reported Hypertension, VIGITEL, 2008

Characteristic	Self-Reported Hypertension, % (95% CI)^a^	All Respondents, % (95% CI)^a^
**Sex**		
Female	26.3 (25.3–27.4)	53.9 (52.8–55.0)
Male	21.0 (19.7–22.3)	46.1 (45.0–47.2)
**Age, y**		
18–24	6.5 (4.8–8.1)	21.5 (20.2–22.8)
25–34	11.2 (9.7–12.6)	25.3 (24.4–26.3)
35–44	21.1 (19.6–22.7)	21.3 (20.5–22.0)
45–54	37.1 (35.1–39.1)	14.6 (14.0–15.2)
55–64	52.1 (49.6–54.6)	8.7 (8.2–9.1)
≥65	61.1 (58.7–63.4)	8.6 (8.2–9.0)
**Education, y**		
0–9	29.9 (28.5–31.4)	55.1 (54.1–56.2)
10–12	15.6 (14.8–16.5)	29.6 (28.7–30.4)
>12	18.1 (16.7–19.4)	15.3 (14.7–15.8)
**Overweight or obese (BMI ≥25 kg/m^2^)**	34.2 (32.8–35.6)	44.2 (43.1–45.3)
**Dyslipidemia**	50.0 (48.0–52.0)	16.8 (16.1–17.5)
**Cardiac event**	73.9 (69.4–78.4)	2.8 (2.5–3.1)
**Diabetes**	70.5 (67.4–73.6)	5.5 (5.1–5.9)
**Sufficiently active during leisure time**	20.6 (18.9–22.4)	15.0 (14.3–15.7)

Abbreviations: VIGITEL, Sistema de Vigilância de Fatores de Risco e Proteção para Doenças Crônicas por Inquérito Telefônico (Telephone-based Surveillance of Risk and Protective Factors for Chronic Diseases); CI, confidence interval; BMI, body mass index.

a Values are weighted to adjust for the sociodemographic distribution of the sample population to the adult population for each city in the 2000 Demographic Census.

The prevalence of self-reported hypertension was high among women, those with less education, and tended to increase with age (Table 1). A third of those who reported having hypertension were overweight or obese. The prevalence of dyslipidemia in those with hypertension was also more than triple the rate of the overall population. Similarly, the rates for a cardiac event and diabetes were higher among those with self-reported hypertension compared with the overall population.

For the 27 cities studied, the total prevalence of LTPA was approximately 15%, 12% for women and 19% for men (Table 2). In the overall population, the rates of LTPA decreased with increasing age, increased with increasing education level, and were higher for men than for women across the sex-specific groups.

**Table 2:** Prevalence of Adults (≥18 years old) Who Reported Practicing Sufficient Leisure-Time Physical Activity^a^, by Age Group and Level of Education (N = 54,353), VIGITEL, 2008

Variable	Sex		Total, % (95% CI)^b^
	Women, % (95% CI)^b^	Men, % (95% CI)^b^	
**Age group, y**			
18–24	9.9 (8.2–11.6)	30.0 (24.0–31.9)	18.6 (16.6–20.5)
25–34	11.6 (10.1–13.1)	18.3 (15.7–20.9)	14.8 (13.3–16.3)
35–44	13.7 (12.2–15.2)	12.8 (11.1–14.4)	13.3 (12.2–14.4)
45–54	13.2 (11.5–14.8)	12.5 (10.7–14.2)	12.8 (11.6–14.0)
55–64	13.0 (11.0–15.1)	18.0 (14.7–21.3)	15.2 (13.4–17.1)
≥65	11.1 (9.2–13.1)	19.3 (15.9–22.8)	14.3 (12.5–16.1)
**Education, y**			
0–9	9.5 (8.5–10.5)	15.0 (13.2–16.8)	12.0 (11.0–13.0)
10–12	14.7 (13.5–15.9)	22.3 (20.7–23.9)	18.1 (17.1–19.1)
>12	15.7 (14.2–17.2)	24.0 (21.7–26.2)	19.6 (18.3–20.9)
**Total**	12.0 (11.3–12.7)	18.5 (17.3–19.7)	15.0 (14.3–15.7)

Abbreviations: VIGITEL, Sistema de Vigilância de Fatores de Risco e Proteção para Doenças Crônicas por Inquérito Telefônico (Telephone-based Surveillance of Risk and Protective Factors for Chronic Diseases); CI, confidence interval.

a Engaged in light or moderate physical activities at least 30 minutes per day on 5 or more days a week, or intense physical activity for 20 minutes per day at least 3 times per week, during leisure time.

b Values are weighted to adjust for the sociodemographic distribution of the sample population to the adult population for each city in the 2000 Demographic Census.

Normal-weight men who practiced sufficient LTPA were less likely than those without sufficient LPTA to report having hypertension (OR, 0.77; 95% CI, 0.63–0.94); the same effect was found for overweight or obese males. We also found no difference in the odds of having self-reported hypertension in women who reported practicing sufficient LTPA and those without sufficient LPTA (OR, 1.03; 95% CI, 0.85–1.24); body weight status also did not change this effect.

## Discussion

Our results demonstrated important differences in the distribution of self-reported hypertension and LTPA according to sociodemographic characteristics; these results are consistent with studies involving data from previous VIGITEL survey years (11,12). The observed higher prevalence of self-reported hypertension among women and older people and people with fewer years of education has been found in other studies (7,11,13). A study of variations by sex and education level involving data from VIGITEL 2007 also found that the prevalence of hypertension was lower among women with more schooling; among men, hypertension rates were lower with increasing years of schooling (11).

A study examining the association between the total amount and intensity of LTPA on the risk of coronary heart disease, hypertension, and diabetes in middle-aged men and women in Finland reported a preventive effect of LTPA on all 3 diseases, but the effects differed by sex (14). An increased total amount of energy expenditure from LTPA and increased intensity of these activities was significantly associated with a reduced risk of hypertension in men but not women. In our study, we also found a weak association between meeting the recommendations for LTPA and the risk of hypertension in women; among men, we found a possible protective effect of LTPA. A possible explanation for these findings is that women tended to report a lower intensity of LTPA and total amount of energy expenditure than men (14).

Overall, sociodemographic characteristics and health status are predictors of physical activity levels and the risk for hypertension and other NCDs. In a sample of Brazilian adults with high blood pressure, less schooling and diabetes were associated with low physical activity levels (15). With respect to predictors of hypertension, a study conducted with adults in Belém, the capital of Pará (Northern Brazil), found that age and overweight were directly associated with hypertension; the risk of hypertension was 6.33 times higher among obese men and 3.33 times higher among obese women than among normal-weight adults (16). In addition, age, education, and food consumption were found to interfere in the association between hypertension and overweight. Our study similarly demonstrated higher mean BMI among those with self-reported hypertension than among those without the condition (data not shown). Prevalence of hypertension was found to be higher among older people and those with less education, groups that also had lower levels of LTPA. Sex and education inequalities in the risk factors for NCDs among Brazilian adults have also been reported (7,11–13).

A possible explanation for the observed differences in the prevalence of self-reported hypertension between men and women is related to health-seeking behaviors. In Brazil, women tend to seek medical care more so than men, which may explain the higher percentage of self-reported chronic disease in women (13).

The low prevalence of LTPA has also been reported in other low- and middle-income countries that conduct national surveillance of physical activity behaviors, including Peru and Mexico (17,18). Differences have been observed between men and women (19,20) in studies conducted in Brazil involving representative samples of the Southeast and Northeast regions (21) and Southern regions (22), in high-income countries such as the United States (23) and Australia (24), and LMICs such as Peru (17).

A limitation to our study is the use of self-reported data for chronic disease status, physical activity levels, and weight and height. In low- and middle-income countries, most population-based studies for NCD-related health behaviors still rely on self-report, which poses a couple of challenges. One of these challenges relates to literacy issues, where low literacy among the sample population can make self-administration of the survey instruments difficult (25). For the Brazilian population, recalling health-related risk behaviors can be challenging if the questionnaire lacks culturally appropriate items, is poorly translated (from the English BRFSS version), or, in terms of physical activity, fails to assess the types of activities in which this population participates. Social desirability bias is another challenge for self-report measures; for example, the population may overreport their physical activity levels because of an increased awareness of the importance of physical activity. Overreporting and underreporting of weight is also commonly observed with self-report tools. Regarding self-report of chronic diseases, the rates for hypertension and diabetes are likely underestimated when compared with surveys conducted with biomedical measurements for diagnosis (26,27), because these conditions can be asymptomatic (ie, numerous respondents may have the condition but not know it without a formal physician diagnosis). Nevertheless, a Brazilian population-based study conducted in a Southeastern city found good sensitivity (72%) and specificity (86%) for self-reporting of hypertension (28). Another study from the United States (29) involving the National Health and Nutrition Examination Survey III (1988–1991) also found that self-reporting for hypertension had good sensitivity (71%) and specificity (92%), suggesting that self-reporting tools can be suitable measures of the prevalence of hypertension in a population. Sensitivity for self-reporting of diabetes, however, is lower than for hypertension as the condition is more complex and less widespread, because a number of people are unaware that they have the disease. The study from the Southeastern city in Brazil that involved an elderly population found a 57.1% sensitivity for self-reported diabetes when compared with a physician diagnosis and fasting glycemia (27). Validity of the data obtained by VIGITEL 2008 has been reported elsewhere (30). Ongoing evaluation of the validity of the VIGITEL survey questions regarding self-reported disease can help quantify the proportion of false positives (resulting from reporting bias). In addition, conducting household surveys involving direct measurements of glycemia and arterial pressure can be costly and complex; thus, the data collected by VIGITEL can be valuable to processes of planning, monitoring, and evaluating national activities for the control and prevention of diabetes and hypertension in Brazil.

We also acknowledge the potential for selection bias of our study population because of the inclusion of participants who may engage in low levels of physical activity because of their health status (ie, having a chronic condition); however, because the survey does not include questions about barriers to physical activity we could not identify people unable to participate in physical activity because they had a chronic condition. Bias in the opposite direction is also possible where the sample includes participants with a chronic condition who report engaging in higher levels of physical activity because they are advised to participate in physical activity to improve their health. Inclusion of questions on barriers and facilitators to physical activity in the survey could help identify the extent to which selection bias is present.

Another limiting factor of this study is the restriction of the sample population to adults living in Brazilian state capitals and the Federal District who own a telephone landline, because of an unequal distribution of telephone coverage in Brazil (13) and increasing ownership and use of cellular telephones over landline phones. The cross-sectional design of the study also does not allow for cause–effect conclusions.

To better understand the complex relationship between LTPA and chronic disease, inclusion of other domains of physical activity into analytic models may be necessary, including physical activity for transport, household chores, and occupation. Physical activity in the household domain may change the VIGITEL data analyses by sex, because women are 3 times more active in this domain than men (6). The interpretation of this physical activity domain, however, is complex because of the lack of evidence of the health benefits of household physical activity. In addition, slight increases in work-related physical activity should be carefully analyzed because trends may depend on the activities performed and the safety and ergonomic quality of the work.

Although efforts to promote LTPA have increased in Brazil, LTPA prevalence recorded by VIGITEL demonstrates minimal changes (6). In addition, few studies have been conducted to evaluate the association between risk factors for NCDs and disease status in a Latin American context. Our findings are novel in this sense and we highlight the differences found in the relationship between LTPA and hypertension when examined by sex; such a finding has not been reported elsewhere. We also demonstrate that the LTPA–hypertension relationship does not differ among those of normal weight and those overweight or obese. These findings may suggest that public health priorities in Brazil aimed at addressing hypertension in adults via LTPA promotion can target the entire population and not just those who are overweight or obese. In addition, although the effect for Brazilian women was found to be nonsignificant, this could be because this subgroup may engage in other non-LTPA activities that contribute more to their overall physical activity, such as household chores. To examine this notion, separate analyses with VIGITEL data can examine the relationship between NCDs and other non-LTPA domains by sex. To obtain a more accurate assessment of this association, use of objective measures of physical activity (eg, use of accelerometry) among a representative sample population are recommended, particularly to reduce potential underreporting or overreporting of physical activity that is commonly found with self-reports. However, costs and time required to conduct large studies using accelerometry would be greater than applying self-report methods, including VIGITEL.

Despite the limitations of telephone-based surveys that may underestimate frequencies of disease or risk factors and may involve false reports from respondents, studies from the United States and Brazil suggest that this method of measurement is valid (7,30,31). The benefits of telephone surveys to obtain fast and low-cost information make VIGITEL a valuable tool to monitor key risk factors for NCDs at the regional level in the adult Brazilian population and to inform NCD prevention and health promotion programs and policies directed at such diseases. Our findings point to differences in the prevalence of hypertension across sociodemographic characteristics such as sex, age, and education level and to the complex influence of sex on the association between LTPA and hypertension. Although our findings cannot support causal inferences, they point to the need for further investigation of the complex relationship between physical activity and NCDs; we also recommend that future studies consider the role of other domains of physical activity in the association between physical activity and these diseases.
